# Coronary Heart Disease and Emotional Intelligence

**DOI:** 10.5539/gjhs.v5n6p156

**Published:** 2013-09-23

**Authors:** Chrisanthy P. Vlachaki, Katerina Maridaki-Kassotaki

**Affiliations:** 1 Department of Home Economics and Ecology Harokopio University, Kallithea, Attica, Greece

**Keywords:** coronary, heart disease, emotion, emotional intelligence, regulation, risk factor

## Abstract

**Background:**

Coronary Heart Disease (CHD) is associated with emotions, especially negative ones, namely anxiety and depression. Emotional Intelligence (EI) is a psychological model that consists of a variety of emotional skills.

**Aims:**

The aim of the present study was to examine the relation between different dimensions of Emotional Intelligence and coronary heart disease.

**Methods:**

A total of 300 participants were studied during a 3-year period in an attempt to partially replicate and further expand a previous study conducted in Greece among CHD patients, which indicated a strong association between certain dimensions of Emotional Intelligence and the incidence of CHD. All participants completed a self-report questionnaire, assessing several aspects of Emotional Intelligence.

**Findings:**

The results showed that there is a link between the regulation of emotions and the occurrence of CHD.

**Conclusions:**

The evidence reported in the present study makes stronger the claim that EI plays a significant role in the occurrence of CHD.

## 1. Introduction

While there is a great amount of interesting scientific research indicating that the autonomic nervous system may be a major component of the emotion response, a simple model that can guide a physician in managing the emotions of a person in favor of his health has not been produced yet (Kreibig, 2010). Numerous studies have found, however, that negative emotions and thoughts may cause disease along with other important risk factors, while positive emotions can improve health and eliminate disease. Most risk factors that drive Coronary Heart Disease (CHD) have genetic, environmental, physiologic and behavioral components (Kones, 2011). The disease can be influenced by negative emotions, such as stress, anger, anxiety, resentment and depression (Das & O’Keefe, 2006; Sirois & Burg, 2003). There is a very significant body of knowledge regarding the relation between health and emotions (Gross, 2013; Kubzansky, 2011). However, this relation has not been thoroughly evaluated within the context of a psychological model.

The present study attempted to partially replicate and further enhance a previous one conducted among fifty-six Greek CHD patients which examined the relationship between understanding of emotions, namely self-emotion appraisal, others’ emotion appraisal, use of emotions and regulation of emotions and coronary heart disease, within the psychological context of Emotional Intelligence (Kravvariti, Maridaki-Kassotaki, & Kravvaritis, 2010). The researchers have demonstrated that self-reported aspects of Emotional Intelligence (EI), such as use of emotions and regulation of emotions, are associated with incidence of coronary heart disease. The dimension “use of emotion” or, in other words, “utilization of emotions” refers to the individual’s ability to utilize and direct emotions in order to improve his performance. A person who is highly capable in this dimension would be able to encourage himself and move towards positive directions. A person who is highly capable in the dimension “regulation of emotion” would have better control of his emotions.

The present study was an attempt to replicate the above study using a greater sample of patients (approximately 3-fold increase in sample size) and an additional number of CHD risk factors, such as hyperlipidemia, diabetes mellitus, and demographic characteristics, such as marital status. The highly significant risk factors for developing coronary heart disease may act as confounders and they had to be accounted for and managed in accordance to the Kravvariti’s study.

Coronary heart disease is a significant public health issue due to its high prevalence and mortality rate (National Institutes of Health, 2012). A number of clinical and experimental studies have indicated that strong emotions, especially negative emotions, such as anger, hostility, depression and stress precipitate coronary heart disease. Coronary heart disease patients, for example, have difficulty in coping with stress and depression and experience negative emotions, like anger or frustration. Positive emotions, on the other hand, especially hope, contribute to health benefits and lead to lower levels of coronary heart disease (Tunstall-Pedoe, 2001; Carr, 2008; Koelsch, Enge & Jentschke, 2012).

Among the negative emotions, stress is one of the most predisposing factors of people with coronary heart disease (Glozier et al., 2013). There are studies showing that stress caused by conflicts in close relationships (family, friends, coworkers, spouses) may predict myocardial infarction for both genders (De Vogli, Chandola & Marmot, 2007). Similarly, stressful marital relationships may triple the risk for recurrent coronary heart disease events (Orth-Gomér et al., 2000; Orth-Gomér & Leineweber, 2005). Further, job strain along with lack of effort-reward policies may cause coronary heart disease for men and women (Kuper & Marmot, 2003; Kuper et al., 2002; Eaker et al., 2004).

Moreover, another negative psychological state such as depression is associated with coronary heart disease (Compare et al., 2013). Numerous studies have confirmed the prominence of depressive symptoms and major depression in patients with coronary heart disease (Hemingway & Marmot, 1999; Panagiotakos et al., 2002; Goldston & Baillie, 2008; Rozanski, Blumenthal, & Kaplan, 1999). Patients with depression have increased platelet reactivity, decreased heart variability and increased inflammatory markers, such as C-reactive protein, which are all risk factors for coronary heart disease (Reichenberg et al., 2001). Further, there is evidence to show that the continued presence of depression after recovery of myocardial infarction increases the risk of death to 17 percent within 6 months after the event (Frasure-Smith, Lespérance, & Talajic, 1993).

Further, anger and hostility have been shown to influence risk of coronary heart disease (Kent & Shapiro, 2009; Chida & Steptoe, 2009). On the other hand, positive emotions, for example optimism is associated with reduced risk for myocardial infarction and coronary heart disease mortality in the Women’s Health Initiative study (Tindle et al., 2009), as well as the frequent expression of positive emotions is associated with decreased incidence of coronary heart disease (Saxena et al., 2012; Low, Thurston, & Matthews, 2010). Increased frequency of positive feelings and decreased frequency of depressive symptoms might be indicated as a preventive factor for coronary heart disease (Davidson et al., 2010). In a recent study, Denollet (2010) indicated that suppression of emotions is a major risk factor for coronary heart disease. He managed to illustrate that cardiac patients who tend to inhibit the expression of their emotions have a higher death rate than other patients (Denollet et al., 1996).

As stated above, there is a great amount of evidence attesting to a link between coronary heart disease and psychological states like depression, anger, anxiety, stress, etc. Little evidence, however, has been provided thus far attesting to the link between coronary heart disease and emotions within a psychological model, like that of Emotional Intelligence proposed by recent theorists (Mayer & Salovey, 1997; Goleman, 1995; Petrides, 2001). In their theories, Emotional Intelligence (EI) has been characterized as *“the ability to perceive emotions, to access and generate emotions so as to assist thought, to understand emotions and emotional knowledge, and to reflectively regulate emotions so as to promote emotional and intellectual growth”*. The term EI was first used in the doctoral thesis of Wayne Payne (1986), entitled: “A Study of Emotion: Developing Emotional Intelligence”, where he defined EI as the ability to express emotions openly. In 1995, Daniel Goleman published his book “Emotional Intelligence: Why It Can Matter More than IQ” and made the term widely used.

There are two different constructs of EI, trait EI and ability EI. Trait EI concerns emotion-related self-perceptions measured by self-report questionnaires and ability EI concerns emotion-related cognitive abilities that ought to be measured by maximum performance questionnaires (Petrides & Furnham, 2001). The maximum performance questionnaire is defined by the work of Mayer and his colleagues in 1999. The later developed items that can be responded to correctly or incorrectly, in contrast to self-reported items (Mayer, Salovey, & Caruso, 1999; Mayer et al., 2003).

The aforementioned study conducted in Greece has been, so far, the only study, which has examined the above issue. The present study was designed to partially replicate the above study in a greater sample of Greek patients. In addition, it sought to examine the relation of a number of atherosclerotic risk factors, as well as demographic characteristics to CHD incidence. The major atherosclerotic risk factors and some demographic characteristics are well-known prognostic factors for the disease. If they are not measured appropriately, they may lead to bias and distort the magnitude of the relationship between Emotional Intelligence and coronary heart disease. For that reason, they were accounted as confounding variables.

Based on previous relevant studies, the hypothesis made in the study was that individuals who suffer from coronary heart disease would have low performance in the tests assessing dimensions of Emotional Intelligence, namely self-emotion appraisal, others’ emotion appraisal, use of emotions and regulation of emotions. Furthermore, it is hypothesized that various dimensions of Emotional Intelligence may be good predictors of coronary heart disease and should be accounted for as potential risk factors, such as smoking or family history of CHD, in future research.

## 2. Method

The data were gathered from the General Hospital of Attica “KAT” in Athens, Greece, during a 3-year period. Written informed consents were obtained from all studied subjects. The study was funded by the Onassis Foundation and approval to be conducted was granted by the Authorities and Scientific board of the above Hospital.

### 2.1 Participants

Three hundred (300) hospitalized patients – 150 with and 150 without CHD - were recruited for participation in this study. CHD patients were hospitalized in the department of Cardiology of the General Hospital of Attica “KAT” and had a documented history of myocardial infarction, or angiographic evidence of coronary artery disease and/or positive treadmill ECG test (Scanlon et al., 1999). Non-CHD patients were assessed free of significant CHD and were hospitalized in the department of Orthopedics of the above hospital. The characteristics of the subjects measured, included demographics, family history of CHD and atherosclerotic risk factors, such as hyperlipidemia, diabetes mellitus, hypertension, smoking, obesity, with the respective data been collected from their medical files. Both groups consisted of hospitalized patients, because they experience more stress, fear of the unknown and embarrassment during hospitalization when compared to a non-hospitalized group of people (Wilson-Barnett, 2008).

### 2.2 Measures

The Greek version of the self-report Wong & Law Emotional Intelligence Scale (WLEIS) was used in order to assess Emotional Intelligence. It consists of 16 items falling into four categories, each category representing a different dimension of Emotional Intelligence and comprising four items. More specifically, the self-emotion appraisal dimension refers to an individual’s self-perceived ability to understand his emotions (e.g., “I really understand what I feel”). The others’ emotion appraisal dimension refers to a person’s tendency to be able to perceive other peoples’ emotions (e.g., “I have good understanding of the emotions of people around me”). The use of emotions dimension concerns the self-perceived tendency to motivate one self to enhance performance (e.g. “I am a self-motivated person”). The regulation of emotions dimension concerns individuals’ perceived ability to regulate and control their own emotions (e.g., “I am quite capable of controlling my own emotions”). Each item was rated on a seven point Likert scale (1 = strongly disagree, to 7 = strongly agree). The validity and reliability of the Greek version of the WLEIS instrument have been established by Kafetsios and Zampetakis (2008). Cronbach Alpha reliability coefficients of the Greek version of the WLEIS factors in the present study were found to be 0.70, 0.71, 0.78, and 0.78 for self-emotion appraisal, others’ emotion appraisal, use of emotions and regulation of emotions, respectively. Although Cronbach’s alpha values of 0.7-0.8 are generally considered acceptable, they still have a 20-30% chance of not measuring the concepts under study and affect the findings. A test with a reliability of 0.7 means that 30% of its variance is made up of errors.

### 2.3 Statistical Analysis

Normality of distribution was assessed using the Kolmogorov-Smirnov test. A power analysis was used to determine the sample size. Comparison between two groups was performed with Student’s t tests or Mann–Whitney U tests, whether they follow the normal distribution or not. Pearson’s Chi-square calculations were used to compare qualitative variables represented as frequencies. Binary logistic regression was used to determine the variables that independently contributed to the presence of CHD. Odds ratio (OR) and 95% confidence interval (CI) were calculated. All tests were two-sided and P < 0.05 was considered statistically significant. Statistical analyses were performed using SPSS 17.0 (IBM SPSS, Inc., Chicago, USA).

## 3. Results

Table 1 shows the demographic characteristics and the atherosclerotic risk factors for the two groups (CHD patients and non-CHD patients). Three hundred (300) hospitalized patients – 150 with and 150 without CHD - participated in this study. The mean age of CHD participants was 69.9 years (SD = 9.37) and 74.7% of them were males. The mean age of non-CHD patients was 69.1 years (SD = 10.86) and 74.7% of them were males. They did not differ reliably from that of CHD patients. Most of the subjects were married (77.3% of CHD patients and 74.7% of non-CHD patients). The prevalence of atherosclerotic risk factors was significantly higher in CHD patients, as expected. Significantly more CHD subjects suffered from hyperlipidemia (p<0.001), diabetes mellitus (p<0.001) and obesity (p=0.011), as compared to non-CHD patients

**Table 1:** Absolute and mean values of demographic characteristics and atherosclerotic risk factors between the two groups, CHD and non-CHD patients (pts)

	CHD pts (n=150)	Non-CHD pts (n=150)	*P-value*
**Age** (years)	69,85 ± 9,73	69,10 ± 10,86	0,708
**Gender** (male)	112 (74,7%)	112 (74,7%)	1,000
**Marital status**			0,494
Married	116 (77,3%)	112 (74,7%)	
Not married	13 (8,7%)	9 (6,0%)	
Widow	16 (10,7%)	24 (16,0%)	
Divorced	5 (3,3%)	5 (3,0%)	
**Risk factors**			
Hyperlipidemia	102 (68,0%)	39 (26,0%)	**<0,001**
Diabetes	44 (29,3%)	18 (12,0%)	**<0,001**
Hypetension	92 (61,3%)	92 (61,3%)	1,000
Family history of CHD	36 (24,0%)	43 (28,7%)	0,359
Smoking	41 (27,3%)	40 (26,7%)	0,897
Obesity	41 (27,3%)	23 (15,3%)	**0,011**

Data are mean ± SD and number (%).

Table 2 presents the performance in each of the four dimensions of the EI test for the two groups. The patients with CHD had significantly lower performance in all the examined dimensions of Emotional Intelligence compared to non-CHD patients (p<0.001). However, no significant difference was observed in hypertension, family history of CHD and smoking.

**Table 2:** Absolute and mean values of performance of patients in each of four dimensions of the Emotional Intelligence (EI) test between the two groups, CHD and non-CHD patients (pts)

	CHD pts (n=150)	Non-CHD pts (n=150)	*P-value*
**Dimensions of Emotional Intelligence (EI)**			
1. Self-Emotion Appraisal	5,55 ± 1,07	6,29 ± 0,74	**<0,001**
2. Others’ Emotion Appraisal	5,40 ± 1,11	6,21 ± 0,72	**<0,001**
3. Use of Emotions	5,32 ± 1,27	6,30 ± 0,68	**<0,001**
4. Regulation of Emotions	4,97 ± 1,22	6,30 ± 0,73	**<0,001**

Data are mean ± SD and number (%).

In order to examine whether emotions may be good predictors of CHD, a binary logistic regression analysis was conducted on the data (see Table 3). In this analysis, the outcome measure was the presence of CHD. The independent variables were the four components of the WLEIS questionnaire and the classical atherosclerotic risk factors, such as hyperlipidemia, diabetes mellitus, hypertension, family history of CHD, smoking and obesity. The well-established risk factors, namely hypertension, family history for CHD and smoking, were included in the model, even though they were not found to have a significant difference in their mean values between the two groups, because they are potential confounders according to the bibliography (Brenner & Blettner, 1997). In order to evaluate the utility of the statistical model, the overall percentage accuracy rate was estimated. The percentage of accuracy was produced by SPSS at the last step in the Classification Table. An accuracy rate of 82.7%, suggests that the model is useful, because it predicts accurately 82.7% of cases.

**Table 3:** Logistic Regression Analyses for the effect of emotional and atherosclerotic risk factors on CHD

	B	Odds Ratio Exp (B)	95% Confidence Interval	*P-value*
**Dimensions of Emotional Intelligence (EI)**				
Self-Emotion Appraisal	0.502	1.652	0.866 – 3.152	0.128
Others’ Emotion Appraisal	-0.522	0.593	0.342 – 1.029	0.063
Use of Emotions	-0.136	0.873	0.501 – 1.521	0.632
Regulation of Emotions	-1.428	0.240	0.138 – 0.416	**<0.001**
**Risk factors**				
Hyperlipidemia	1.947	7.011	3.625 – 13.560	**<0.001**
Diabetes	1.249	3.485	1.520 – 7.990	**0.003**
Hypertension	0.187	1.206	0.620 – 2.345	0.582
Family history of CHD	-0.299	0.742	0.351 – 1.569	0.434
Smoking	0.483	1.621	0.791 – 3.321	0.187
Obesity	0.418	1.519	0.703 – 3.282	0.288

The Cox & Snell R Square and the Nagelkerke R Square values provide an indication of the amount of variation in the dependent variable. These are described as pseudo R square. These values are 0.433 and 0.578 respectively, suggesting that between 43.3% percent and 57.8% percent of the variability is explained by this set of variables used in the model.

As presented in Table 3, regulations of emotions was significant at the 0.001 level thus showing decrease in the odds of the disease by 76% for one unit increase in the relevant score. Patients with lower ability to regulate their emotions have about 76% higher likelihood of suffering from CHD (odds ratio = 0.240, p < 0.001). Participants with hyperlipidemia have 601.1% higher likelihood of suffering from CHD (odds ratio = 7.011, p <0.001). Furthermore, participants with diabetes mellitus have 248.5% higher likelihood of being diagnosed with CHD (odds ratio = 3.485, p = 0.003). Self-Emotion Appraisal, Others’ Emotion Appraisal, Use of Emotion, hypertension, family history of CHD and smoking and obesity did not appear to play a part in the occurrence of the disease, since they failed to reach significance (p-value > 0.05). Therefore, Regulation of Emotions, hyperlipidemia and diabetes mellitus are significant predictors of CHD.

## 4. Discussion

The purpose of this study was to examine the relationship between coronary heart disease and four dimensions of Emotional Intelligence, namely self-emotion appraisal, others’ emotion appraisal, use of emotions and regulation of emotions. Most importantly, the present data are congruent and expand evidence reported in a previous study conducted in Greece, which has established the relation between coronary heart disease and regulation of emotions (Kravvariti, Maridaki-Kassotaki, & Kravvaritis, 2010). In the present study, as mentioned above, the researchers attempted to replicate these findings and indicated that decreased ability to regulate emotions is associated with the incidence of coronary heart disease.

Moreover, the obtained data suggest that people with CHD may suffer from hyperlipidemia and diabetes too. These results are in line with numerous previous studies showing that patients with diabetes have higher risk of developing CHD than people without diabetes (Bays, 2012; Zhang et al., 2012; Sousa et al., 2011). According to several studies, the prevalence of hyperlipidemia in CHD patients is higher compared to that of age-matched controls without CHD (Simon, 2012; Watkins, 2004). The findings of numerous studies support that there is a negative relationship between increased levels of Emotional Intelligence and physical health (Tsaousis & Nikolaou, 2005; Martins, Ramalho & Morin, 2010; Schutte et al., 2007). However, there is no evidence to indicate a link between Emotional Intelligence and hyperlipidemia or diabetes mellitus.

The core finding of the present study was that the regulation of emotions is a good predictor of coronary heart disease. More specifically, the occurrence of coronary heart disease is related to decreased ability to regulate emotions. In the present study, the regulation of emotions reflected the ability to control temper and one’s emotions as well as the ability to handle with difficulties and one’s anger. The reported evidence, therefore, shows that coronary heart disease patients have difficulty in controlling their anger and temper, as well as their emotions. The present findings are in line with previous evidence showing that specific negative emotions such as anger inhibition may increase the risk of coronary heart disease, whereas control of emotions may be protective (Graves, 1994; Harburg, 2003). They also support the claim that anger, hostility or other uncontrolled negative emotions, may cause damage to the cardiovascular system through physiological alterations and increase the risk of a cardiac event (Haines, Cooper, & Meade, 2001; Krantz & McCeney, 2002; Steptoe, 2000).

Previous research support that there is a link between coronary heart disease and Type A behavior pattern (Kent & Shapiro, 2009). People with type A behavior seem to be hostile, aggressive, impatient and competitive. The present findings relate to the old Type A theories, as they reflect that coronary heart disease patients have low ability of regulation of emotions and experience uncontrolled anger and temper.

By and large, the present findings expand previous evidence showing that coronary heart disease patients have difficulties in regulating their emotions. In relation to this, relevant evidence shows that forms of emotion regulation such as reappraisal of emotions and suppression of emotions increases or decreases the levels of C-reactive protein respectively, the C-reactive protein being an inflammatory marker and one of the most well-established risk factors for coronary heart disease (Gross, 2013). In a recent study conducted on more than one thousand participants who were followed up for 13 years, it was found that the successful regulation of emotions is related to decreased risk for coronary heart disease, even when controlling for traditional coronary risk factors (Kubzansky, 2011). Similarly, several other studies have demonstrated that emotion regulation may promote cardiovascular health, reduce the risk for coronary heart disease and contribute to greater well-being (Côté, 2010; Kubzansky & Thurston, 2007; Rozanski et al., 2005; Pennebaker, 1995; Urry & Gross, 2010; Cornelius, 2001; Bushman, 2002).

## 5. Limitations of the Study

The first and most obvious limitation of the present study is the fact that there was used a self-reported measurement of Emotional Intelligence. It is possible that it mirrored the relationship between perceived Emotional Intelligence (not objective) and coronary heart disease. However, even so, it is still an important finding. Additionally, many of the studies cited above appear to have several confounding variables that are likely to affect the findings. This represents a methodological flaw in some studies. The same applies to the current study. The major atherosclerotic risk factors and some demographic characteristics are well-known prognostic factors for the disease. If they are not measured appropriately, they may lead to bias and distort the magnitude of the relationship between Emotional Intelligence and coronary heart disease. The well-established risk factors, namely hypertension, family history for CHD and smoking, were included in the model, even though they were not found to have a significant difference in their mean values between the two groups, because they are potential confounders according to the bibliography. Other risk factors that were not taken into account may have influenced the findings. Finally, the alpha scores only indicate acceptable reliability. They have a 20-30% chance of not measuring the concepts under study and affect the findings.

## 6. Conclusions

A particularly interesting finding of the present study was that a dimension of Emotional Intelligence, namely regulation of emotions, is a good predictor of coronary heart disease. CHD patients have lower abilities of understanding, using and regulating emotions, compared to non-CHD patients, which eventually have an impact on their health. The study of Kravvariti and colleagues was reproduced as closely as possible in a greater sample of patients and was further enhanced with additional risk factors and demographic characteristics. Based on the present findings, we may claim that emotion regulation plays a determinant role in attaining cardiovascular health. Further research, however, is needed in order to identify which emotion regulation strategies (Gross, 1998) are associated with the occurrence of coronary heart disease.

## 7. Implications of the Findings

The present findings may have several practical implications for both research and health care settings. The fact that a dimension of Emotional Intelligence predicts coronary heart disease demands that health care providers should begin to develop programs to foster Emotional Intelligence in CHD patients. If, for instance, patients with coronary heart disease are trained to regulate successfully their emotions with the use of appropriate training programs, their health may be improved. As Emotional Intelligence is teachable and learnable (Goleman, 1998), cardiologists and psychologists should endeavor to teach the principles of Emotional Intelligence to patients. The five strategies, for example, of emotion regulation proposed by Gross (1998) may be used to develop such a program. Future research should focus on the examination of the effectiveness of such training programs.
